# High-angular resolution diffusion imaging generation using 3d u-net

**DOI:** 10.1007/s00234-024-03282-6

**Published:** 2024-01-18

**Authors:** Yuichi Suzuki, Tsuyoshi Ueyama, Kentarou Sakata, Akihiro Kasahara, Hideyuki Iwanaga, Koichiro Yasaka, Osamu Abe

**Affiliations:** 1 Radiology Center, The University of Tokyo Hospital, Tokyo, Japan; 2 Department of Radiology, The University of Tokyo Hospital, Tokyo, Japan

**Keywords:** Diffusion-weighted imaging, High angular resolution diffusion imaging, Artificial intelligence, Orientation distribution function, Tractography

## Abstract

**Purpose:**

To investigate the effects on tractography of artificial intelligence-based prediction of motion-probing gradients (MPGs) in diffusion-weighted imaging (DWI).

**Methods:**

The 251 participants in this study were patients with brain tumors or epileptic seizures who underwent MRI to depict tractography. DWI was performed with 64 MPG directions and b = 0 s/mm^2^ images. The dataset was divided into a training set of 191 (mean age 45.7 [± 19.1] years), a validation set of 30 (mean age 41.6 [± 19.1] years), and a test set of 30 (mean age 49.6 [± 18.3] years) patients. Supervised training of a convolutional neural network was performed using b = 0 images and the first 32 axes of MPG images as the input data and the second 32 axes as the reference data. The trained model was applied to the test data, and tractography was performed using (a) input data only; (b) input plus prediction data; and (c) b = 0 images and the 64 MPG data (as a reference).

**Results:**

In Q-ball imaging tractography, the average dice similarity coefficient (DSC) of the input plus prediction data was 0.715 (± 0.064), which was significantly higher than that of the input data alone (0.697 [± 0.070]) (*p* < 0.05). In generalized q-sampling imaging tractography, the average DSC of the input plus prediction data was 0.769 (± 0.091), which was also significantly higher than that of the input data alone (0.738 [± 0.118]) (*p* < 0.01).

**Conclusion:**

Diffusion tractography is improved by adding predicted MPG images generated by an artificial intelligence model.

**Supplementary Information:**

The online version contains supplementary material available at 10.1007/s00234-024-03282-6.

## Introduction

Diffusion-weighted imaging (DWI) is a unique type of MRI able to non-invasively obtain in vivo information. It is known for its excellent ability to detect acute cerebral infarction, especially when combined with apparent diffusion coefficients (ADCs), which can be acquired with DWI. Thus, it has become an indispensable sequence in clinical practice. Diffusion tensor imaging (DTI) was devised as an application of DWI that uses fractional anisotropy (FA) to obtain typical quantitative values [1]. It is used in various clinical and research settings. Diffusion tensor tractography (DTT) depicts bundles of white matter in the brain by tracing eigenvectors. It is used in various clinical situations, such as preoperative planning for brain tumors, postoperative functional evaluations, brain development evaluations, and tracking the course of degenerative diseases [2–6]. However, DTI and DTT approximate diffusion information within voxels to ellipsoids, making the expression of cross-fibers difficult. High-angular resolution diffusion imaging (HARDI) addresses this by representing cross-fiber regions [7]. Based on this approach, other methods have been developed to increase the amount of angular information using motion-probing gradients (MPG). These include Q-ball imaging (QBI) [8], constrained spherical deconvolution (CSD) [9], and generalized q-sampling imaging (GQI) [10]. Using these HARDI-based techniques has enabled the identification of complex white matter pathways at the junction of the auditory radiation and inferior longitudinal fasciculus [11]. QBI tractography has been found more reliable than DTT in identifying the motor language cortex [12] and the brain origins of stroke. CSD tractography has been shown to have better visualization ability than DTI and its clinical usefulness is high [13].

In recent years, the application of machine learning and deep learning technologies to MRI has progressed. A variety of uses of artificial intelligence (AI) in MRI have been reported, including noise removal [14], brain tumor differentiation [15], contrast agent reduction [16], and cerebral aneurysm detection [17]. Some of these have begun to be used in clinical settings. Of course, the application of AI to DWI is also popular. Specifically, there have been reports on noise and artifact removal [18, 19], super-resolution [20], diffusion profile evaluation [21], tractography [22], and white matter extraction [23]. Using AI in this way, it has become possible not only to improve image quality but also to support diagnoses. However, in recent years, DWI analysis and imaging conditions have become more complex. There has been an increase in analytical methods that require a single b-value but many MPGs [8–10] or multiple b-values, such as diffusion kurtosis imaging [24], neurite orientation dispersion and density imaging [25]. Therefore, imaging time using DWI tends to be longer than that for DTI. It is possible to shorten imaging time using multistage simultaneous excitation technology such as multiband echo planar imaging (EPI) [26–28] and simultaneous multi-slice (SMS) [29–31] but there is a trade-off, with poorer image quality due to insufficient imaging data [32]. Therefore, the reduction of imaging time in DWI using these techniques is limited.

Until now, AI has primarily been used in this field to improve aspects of the acquired images, such as image quality, or to increase the precision with which diseases can be differentiated, as mentioned above. In this study, we took a completely different approach, with the aim of having AI generate predicted DW images. We investigated the extent to which it is possible to shorten scan time and ensure image quality (tractography accuracy) by generating images that would usually be acquired with MRI using AI. We evaluate HARDI data, including AI-generated images using several quantitative parameters, and examine the utility of this approach.

## Materials and Methods

### Participant data

Participants in this study were 251 cases that had already been imaged to depict QBI tractography as an in-hospital clinical examination before brain tumor resection, gamma knife surgery, or epileptic seizure surgery as retrospective data. These inspections were conducted from February 2016 to March 2021.

The analysis of these images analysis was performed using a protocol approved by the Ethics Committee of the University of Tokyo. All procedures were performed in accordance with the tenets of the 2013 revision of the Declaration of Helsinki. The informed consent requirement was waived by the Ethics Committee due to the retrospective nature of the study.

### Imaging parameters and preprocessing

All MR images were acquired using a 3T clinical scanner (MAGNETOM Skyra, Siemens Healthcare, Erlangen, Germany; 45 mT/m max. gradient strength, 200 mT·m^−1^·s^−1^max. slew rate) and a commercial 20-channel matrix head coil. The DWI parameters for single-shot EPI were: repetition time/echo time = 8900/89 ms, in-plane acceleration factor (generalized auto-calibrating partially parallel acquisition factor) = 2, slice thickness/gap = 2.5/0 mm, number of slices = 60, field-of-view = 240 × 240 mm^2^, matrix = 96 × 96, voxel size = 2.5 × 2.5 × 2.5 mm^3^, b = 3000 s/mm^2^, multiple MPG = 64 directions and a single b = 0 s/mm^2^ image using phase-encoding directions along the anteroposterior axis, and scan time = 625 s. We also obtained b0 s/mm^2^ images with reversed phase-encoding directions along the posteroanterior axis for the distortion correction process. In preprocessing, we performed noise removal [33–35] and ringing artifact removal [36, 37] using MRtrix3 [38], distortion and motion correction using FSL top-up [39] and eddy [40], and then B1 field correction [41] using MRtrix3. And 3D T1-weighted image for anatomical image parameters were: repetition time/echo time = 1900/3.16 ms, slice thickness = 1.25 mm, number of slices = 128, field-of-view = 240 × 240 mm^2^, matrix = 192 × 192, voxel size = 1.25 × 1.25 × 1.25 mm^3^, Inversion time; 962ms, and scan time = 206 s.

### Convolutional neural network

**Fig. 1:**
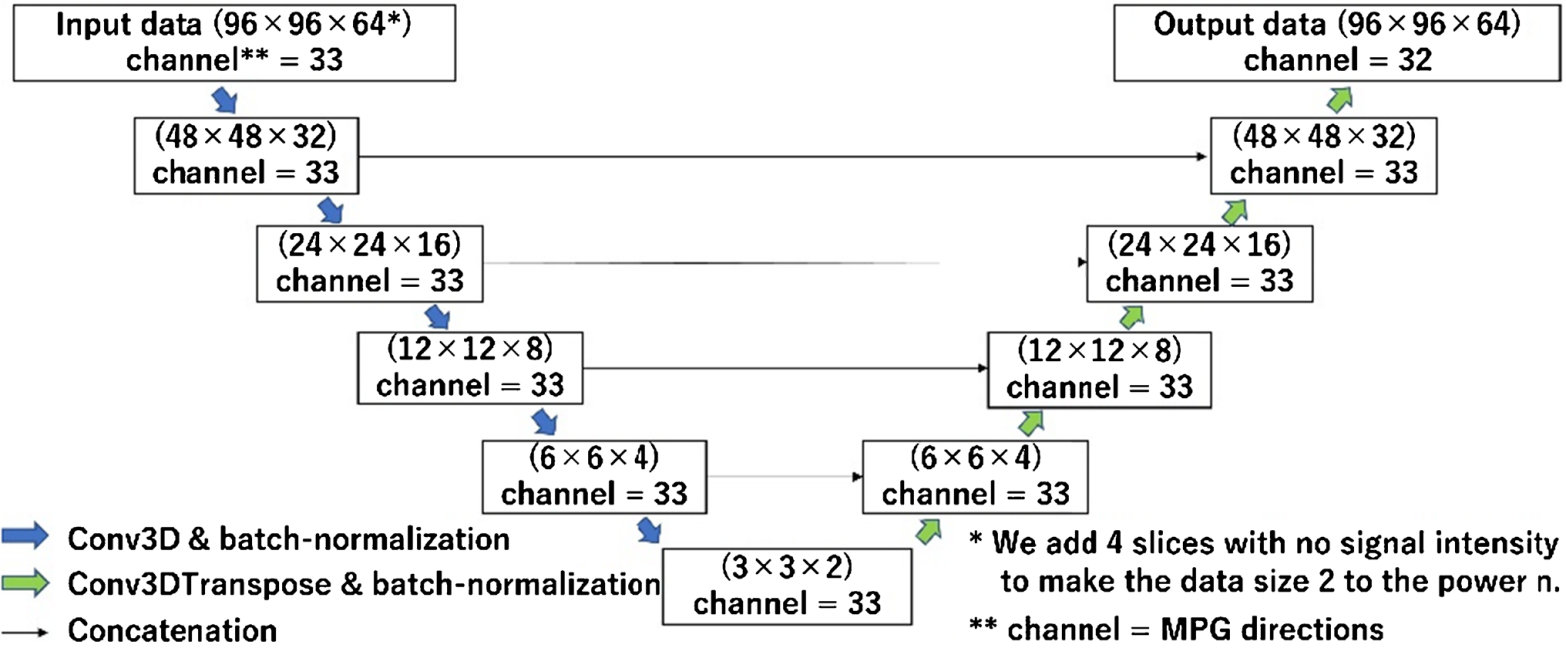
Pipeline depiction of our artificial intelligence imaging generation method using 3D U-net.  MPG, motion-probing gradient

Our dataset was divided into training (n = 191), validation (n = 30), and test (n = 30) sets. We employed a 3D U-net learning model (Fig. 1) created using Python 3.9.7 (https://www.python.org/) and tensorflow-gpu 2.8.0 (https://www.tensorflow.org/) on a computer equipped with a Core i9-12900F central processing unit, 128 GB of random access memory, and a GeForce RTX 3090 graphic processing unit. We used the b0 image and the first 32 axes of HARDI data as input data. The remaining 32 axes of HARDI data were used as the teaching data, and the MPG32 axes output was used as the prediction data. Table 1 shows the MPG array using the electrostatic repulsion method [42] in this study.

**Table 1:**
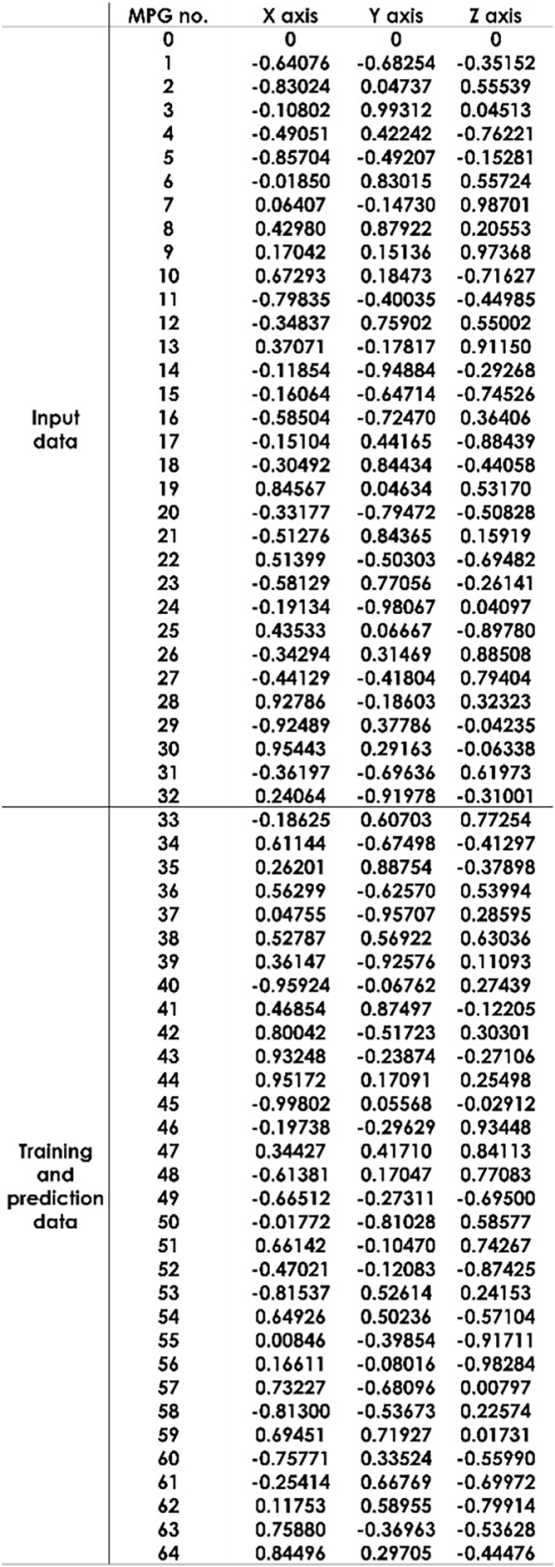
The MPG array using the electrostatic repulsion method

MPG is motion-probing gradient and MPG no.0 indicates b0 image

At the time of learning, we calculated the loss function as the mean squared error (MSE) between the teaching and prediction data. We used Adam as the optimizer. The hyperparameters were optimized using the validation data. The hyperparameters for the 3D U-net model were: kernel size, 3; stride, 2; activation function, rectified linear unit. Those for the supervised learning process were: minibatch size, 15; number of epochs, 300. The trained model was applied to the test data to obtain the predicted data.

### Image evaluations

First, the structural similarity (SSIM) of the prediction data was calculated for each MPG axis, with the latter half of the reference data as the gold standard. We calculated the SSIMs with the multiSSIM3 function of MATLAB 2020b. And we also calculated the peak signal to noise ratio (PSNR). Next, we calculated the orientation distribution function (ODF) and the similarity of the data for the first 32 MPG axes only (input data). The ODF of the reference data and the ODF of the input plus prediction data were compared. ODF calculations were performed using the Diffusion toolkit (https://trackvis.org/dtk/), and quantitative value comparisons were performed using Jensen-Shannon divergences (JSDs) and angular correlation coefficients (ACCs) [43]. JSDs were used to quantify the similarities between two fiber orientation distributions (FODs) or ODFs. We projected both ODFs onto 181 values distributed equally over a sphere. JSD was defined as:

$${\text{JSD}}(P, Q) = \frac{{D}_{KL}\left(P,M\right)+{D}_{KL}(Q,M)}{2}$$

$$\mathrm{M }(i) = \frac{P\left(i\right)+Q (i)}{2}$$

where P(i) and Q(i) are the magnitudes of the histological and MRI FODs (or ODFs) along index i (i = 1 …181), and D_KL_ is the Kullback–Leibler divergence:

$${D}_{KL}(P,Q) = \sum\limits_{i}P\left(i\right){\text{log}}(\frac{P(i)}{Q(i)})$$

We calculated the correlation of functions over a sphere given the spherical harmonic (SH) expansions of both functions. Given two spherical functions and their SH expansions,

$$U(\theta , \varphi ) = {\sum }_{l = 0}^{\infty }{\sum }_{m = -l}^{l}{u}_{lm}{Y}_{lm}(\theta ,\varphi )$$

$${\text{V}}(\theta , \varphi ) = {\sum }_{l = 0}^{\infty }{\sum }_{m = -l}^{l}{v}_{lm}{Y}_{lm}(\theta ,\varphi )$$

the ACC of the functions is calculated as:

$$ACC = \frac{{\sum }_{l = 1}^{\infty }{\sum }_{m = -l}^{l}{u}_{lm}{{v}^{*}}_{lm}}{{[{\sum }_{l{\prime} = 1}^{\infty }{\sum }_{m{\prime} = -l{\prime}}^{l{\prime}}|{{u}_{{l}{\prime}m{\prime}}|}^{2}]}^{{~}^{1}\!\left/ \!{~}_{2}\right.}{{\sum }_{l{\prime}{\prime} = 1}^{\infty }{\sum }_{m{\prime}{\prime} = -l{\prime}{\prime}}^{l{\prime}{\prime}}|{{v}_{{l}^{{\prime}{\prime}}m{\prime}{\prime}}|}^{2}|}^{{~}^{1}\!\left/ \!{~}_{2}\right.}}$$

The calculation areas for the SSIM, JSD, and ACC were masked using the b0 image.

**Fig. 2:**
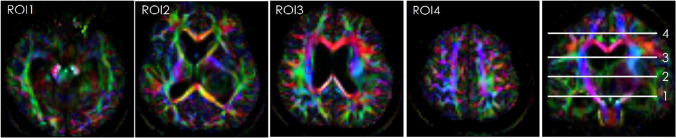
ROI for measurement FA and E1

We performed diffusion tensor analysis and generated FA and color FA from the eigenvalues in our software. In addition, regions of interest (ROIs) were set at the cerebral peduncle (ROI1), posterior peduncle of the internal capsule (ROI2), corona radiata (ROI3), and semiovale center (ROI4) in the normal side on the color FA map. The subjects for ROI measurement were 24 patients, excluding 6 patients with bilateral disease, brain stem disease, and multiple metastases. And the average value and standard deviation (SD) for FA and eigenvalue1 (E1) were calculated. The ROIs were manually set with the consent of one radiological technologist (17 years of MRI experience) and one radiologist (Fig. 2).

Red ellipsoids indicate ROIs in each axial section on the color FA map. ROI1 indicates the cerebral peduncle, ROI2 indicates posterior peduncle of the internal capsule, ROI3 indicates corona radiata and ROI4 indicates semiovale center in the normal side.

Finally, left and right corticospinal tract (CST) and frontal aslant tract (FAT) with Q-ball imaging tractography and generalized q-sampling imaging tractography were visualized, and dice similarity coefficients (DSCs) were calculated for the reference data.

**Fig. 3:**
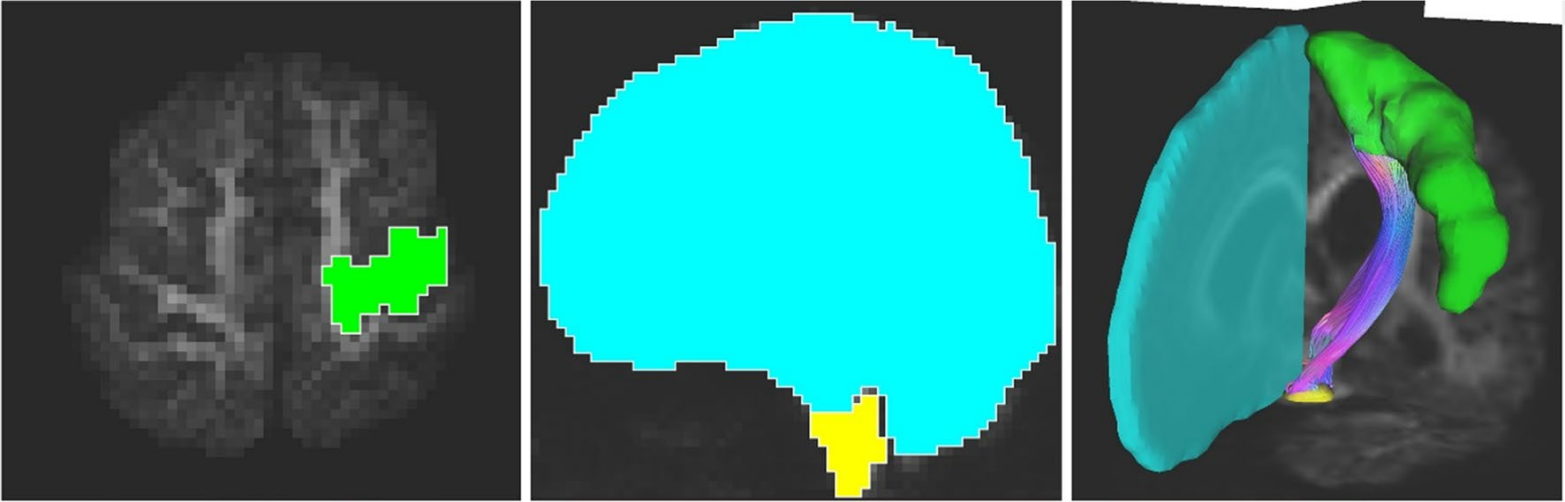
ROIs for depicting CST

QBI tractography was performed using TrackVis (https://trackvis.org/), and GQI tractography was performed using DSI Studio (https://dsi-studio.labsolver.org/). ROIs of CST were automatically obtained by TractSeg [23] analysis. The seed ROI was the cerebral peduncle, with the primary motor cortex as the target point. The midsagittal section was manually set as the avoidance ROI (Fig. 3).

Yellow indicates the cerebral peduncle for seed point, green indicates the primary motor cortex for target point, and blue indicates the midsagittal section for avoidance ROI.

**Fig. 4:**
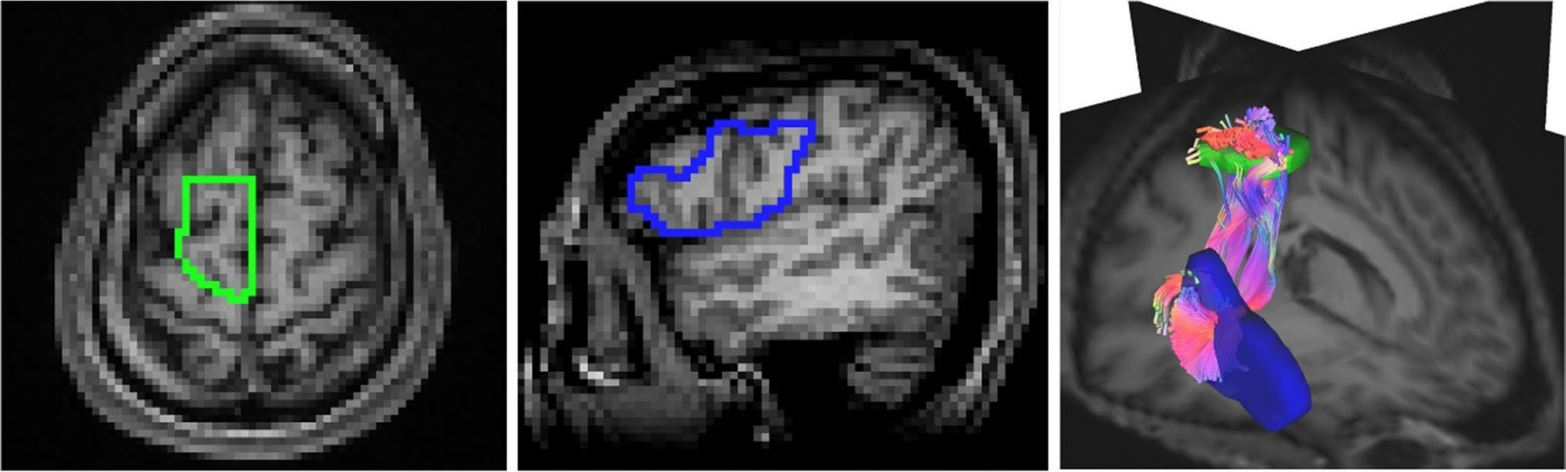
ROIs for depicting FAT

The ROIs for FAT was manually set with the consent of one radiological technologist (17 years of MRI experience) and one radiologist. T1-weighted image was registered to the b0 image using FMRIB's Linear Image Registration Tool; FLIRT (https://fsl.fmrib.ox.ac.uk/fsl/fslwiki/FLIRT). The seed point is (supplementary motor area; SMA and pre-SMA), target point is set in the gill cover of inferior frontal gyrus (IFG), the triangular part of IFG, and the orbital part of IFG [44] (Fig. 4).

Green area indicates the SMA and pre-SMA for seed point, blue area indicates the gill cover of inferior frontal gyrus (IFG), the triangular part of IFG, and the orbital part of IFG.

The default conditions in each piece of software were used for settings such as the tracking algorithms. After voxelization of the corticospinal tracts, we calculated the DSC [45] The DSC as:

$$DSC = \frac{2*V(x\cap y)}{V\left(x\right)+V(y)}$$

where *x* is a corticospinal tract voxel from the reference data, *y* is the corresponding voxel from a fiber bundle generated using input or input plus precision data and *V* is the volume of the relevant voxel.

### Statistics

Post hoc analysis with Freidman tests was conducted with a Bonferroni correction (p < 0.0167) applied to the FA and E1.The Wilcoxon signed-rank test (p < 0.05) was applied to the JSD, ACC, and DSC data.

Statistical analyses were performed using EZR version 4.3.1 software (https://www.jichi.ac.jp/saitama-sct/SaitamaHP.files/download.html).

Unless otherwise specified, data were presented as mean ± SD. Effect size [46] was also calculated for each test.

## Results

### Patient backgrounds

Tables 2 and 3 shows the medical conditions of the participants in each data group. The average ages (± SD) of the patients in the training, validation, and test sets were 45.7 (± 19.1), 41.6 (± 19.1), and 49.6 (± 18.3), respectively.

**Table 2:**
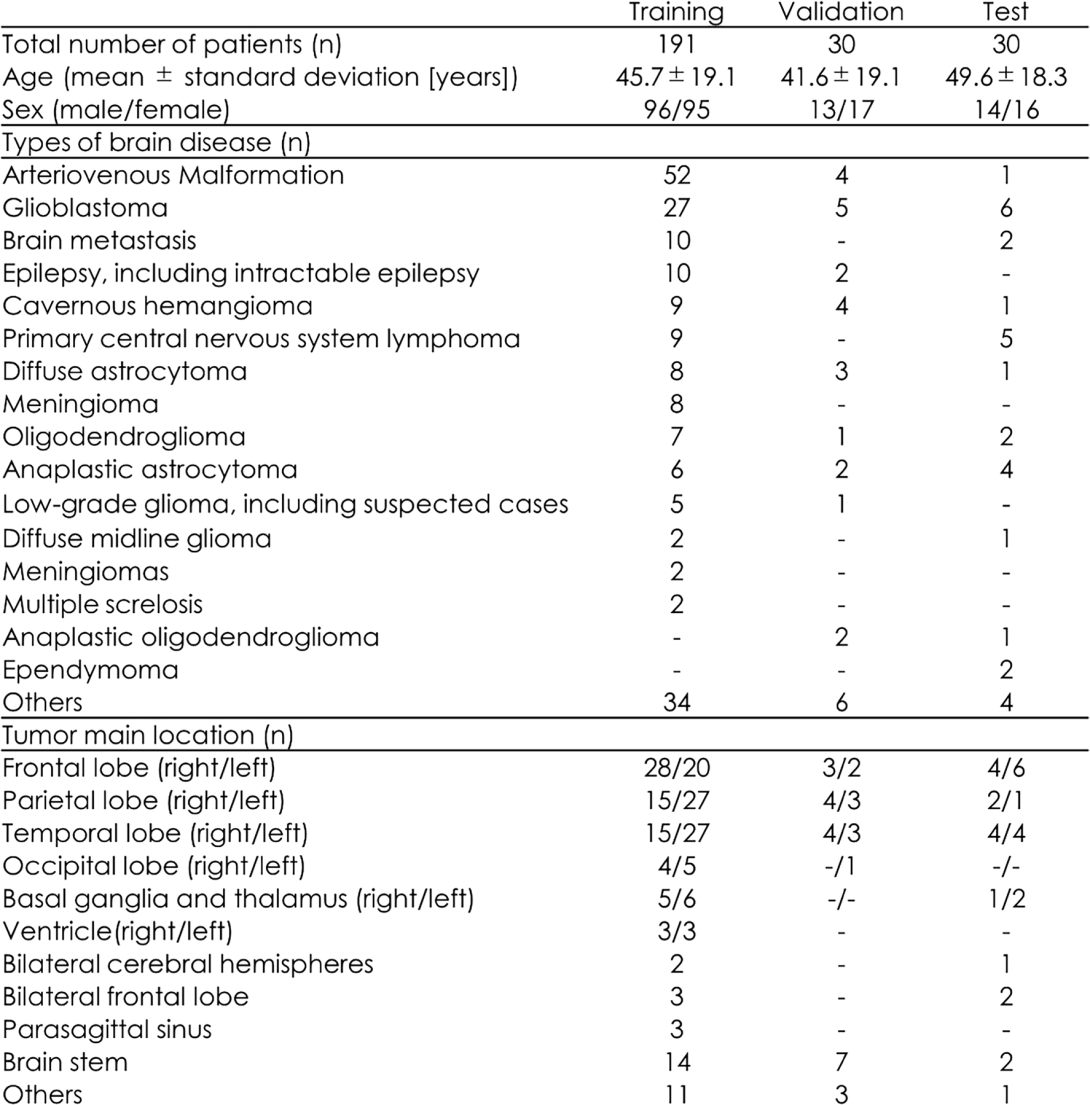
Patient conditions in the three groups

**Table 3:**
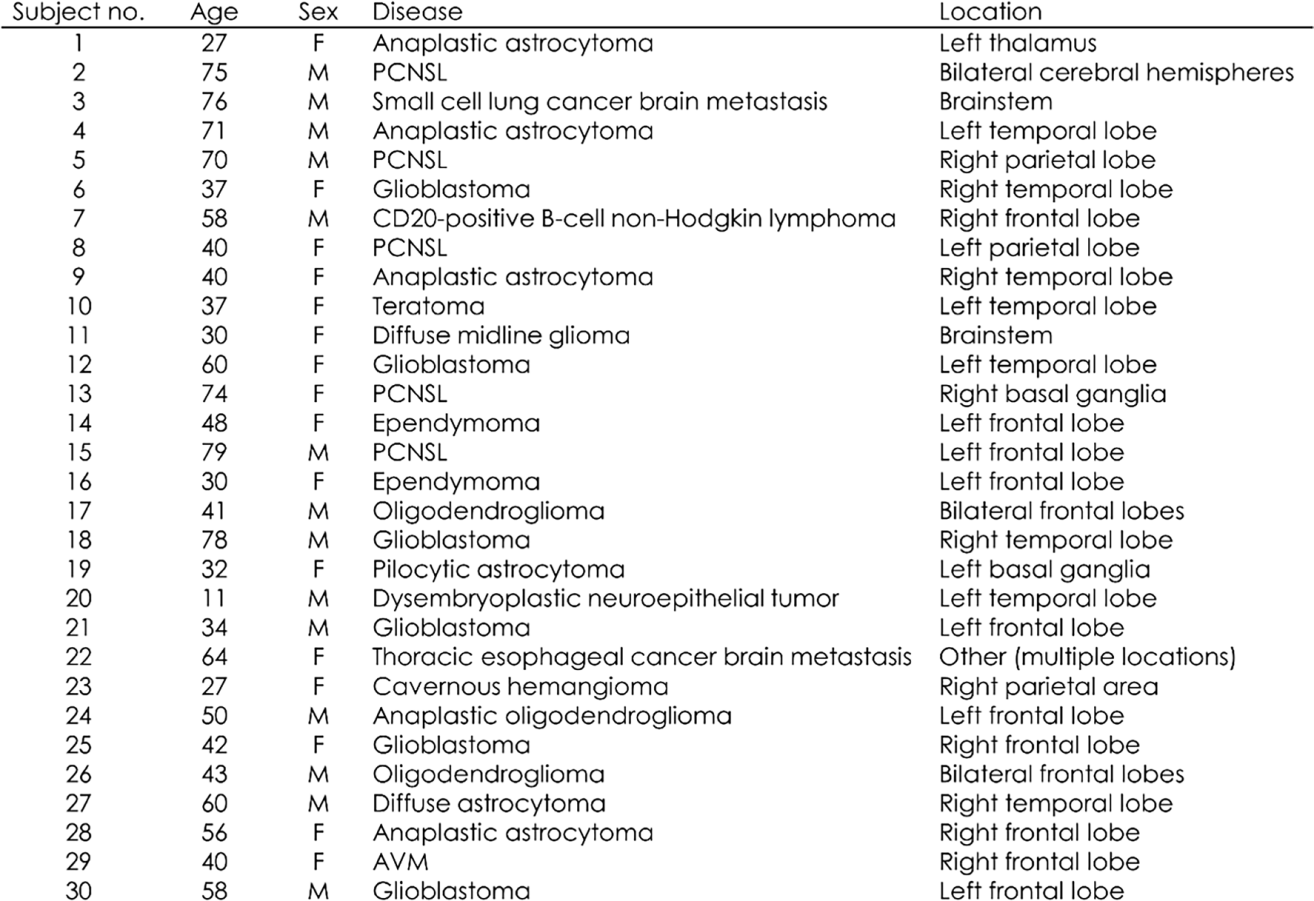
Patient detail conditions in the test data

For brain diseases, cases with only one patient in the training data were classified as “other.” Similarly, when there was only one case with a given tumor location, it was classified as “other.”

Tumor location and brain disease types with no relevant data or numerical values were marked with a hyphen (-) (Table 2).

### Training and validation of the convolutional neural network

**Fig. 5:**
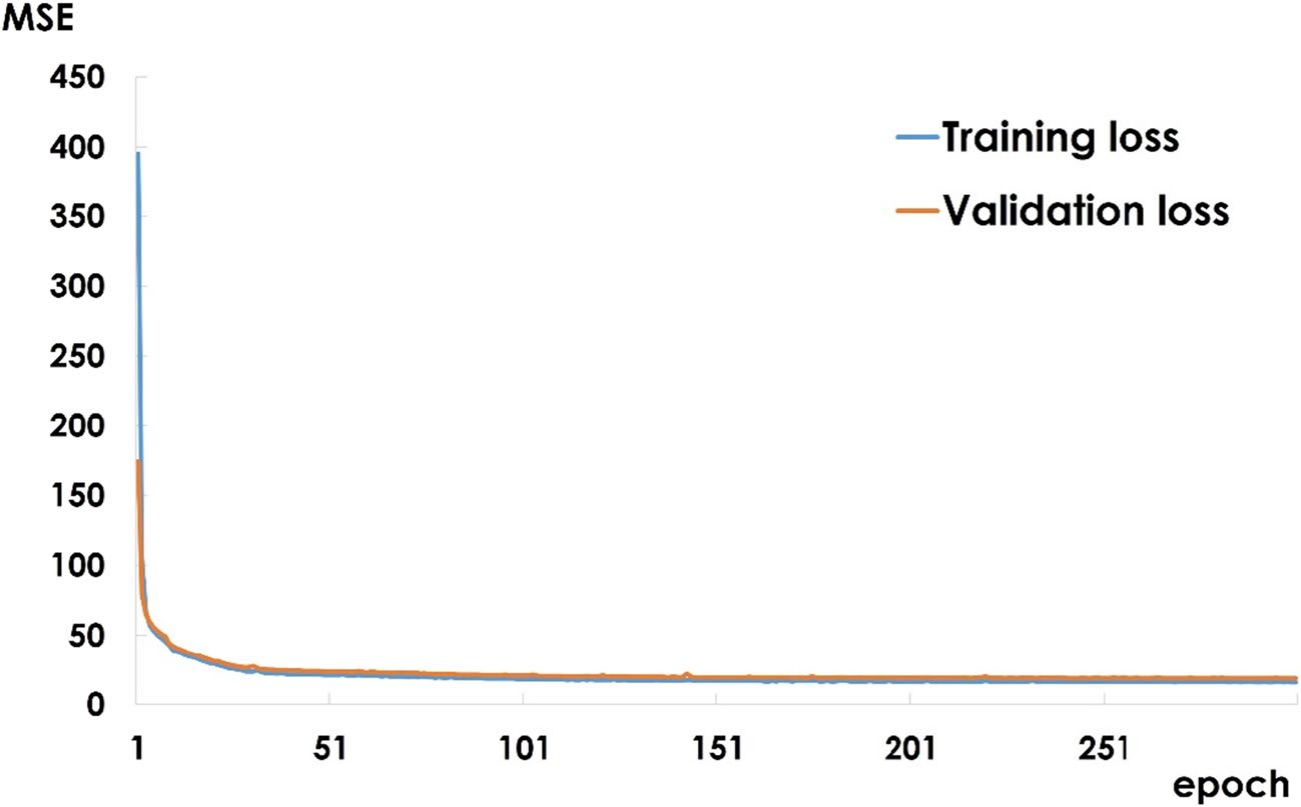
Loss function results

The MSE values from the training process are shown in Fig. 5. The values for the training and validation data after 300 epochs were 15.935 and 18.613, respectively.

MSE, mean squared error.

### DWI similarity evaluation using the test data

**Fig. 6:**
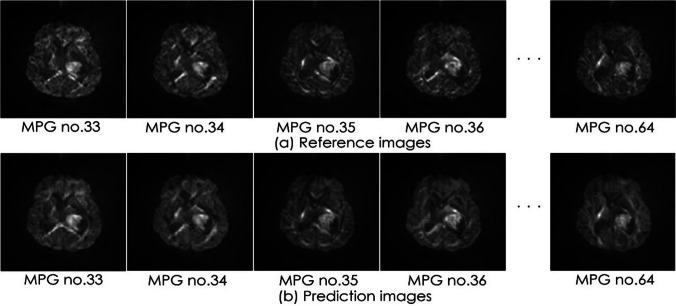
Comparison of prediction and reference images from an example case

The average SSIM for the test data was 0.964 (± 0.010). The minimum and maximum SSIMs for all data were 0.913 and 0.982. The average PSNR for the test data was 35.41 dB (± 1.59). The minimum and maximum PSNRs for all data were 30.36 and 38.32 (Suppl. data). Image comparisons were made between the reference data and predicted data. For each MPG, the predicted image had similar anisotropic contrast to the reference image (Fig. 6).

The case shown is a 27-year-old female with an astrocytoma in the left thalamus.

For each MPG, the prediction image had the same anisotropic contrast as the reference image.

MPG, motion-probing gradient.

**Fig. 7:**
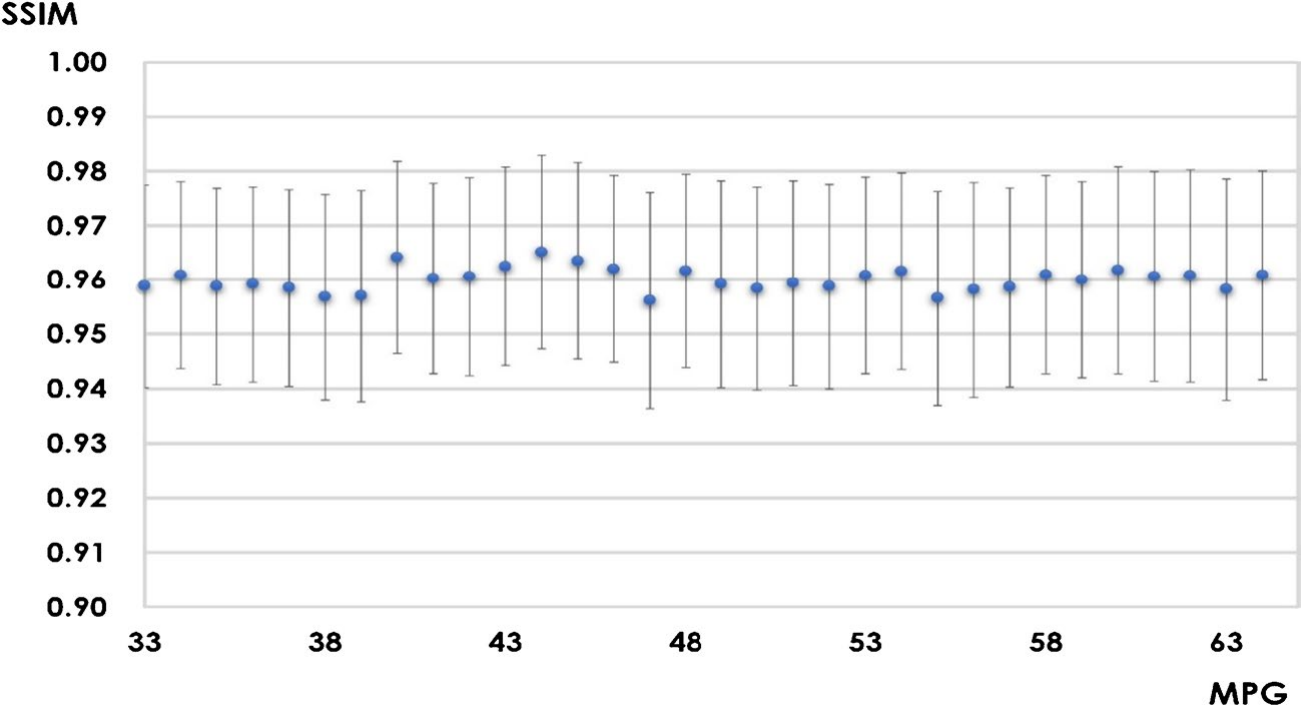
Structural similarities in each motion-probing gradient

There were no obvious outliers from the trend in the average SSIM of each MPG (Fig. 7).

The blue dots represent the average structural similarities. The error bars represent the standard deviations for each motion-probing gradient.

MPG, motion-probing gradient; SSIM, structural similarity.

### Diffusion profile evaluation of the test data

**Fig. 8:**
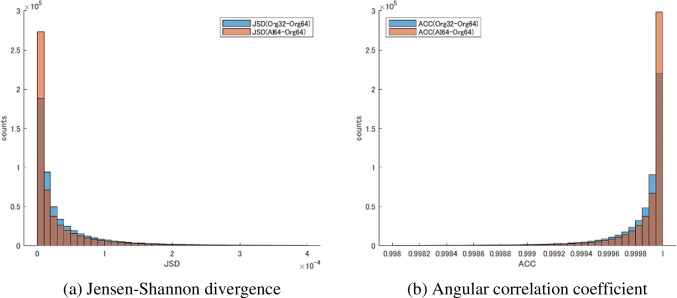
JSD and ACC distributions in an example case

**Fig. 9:**
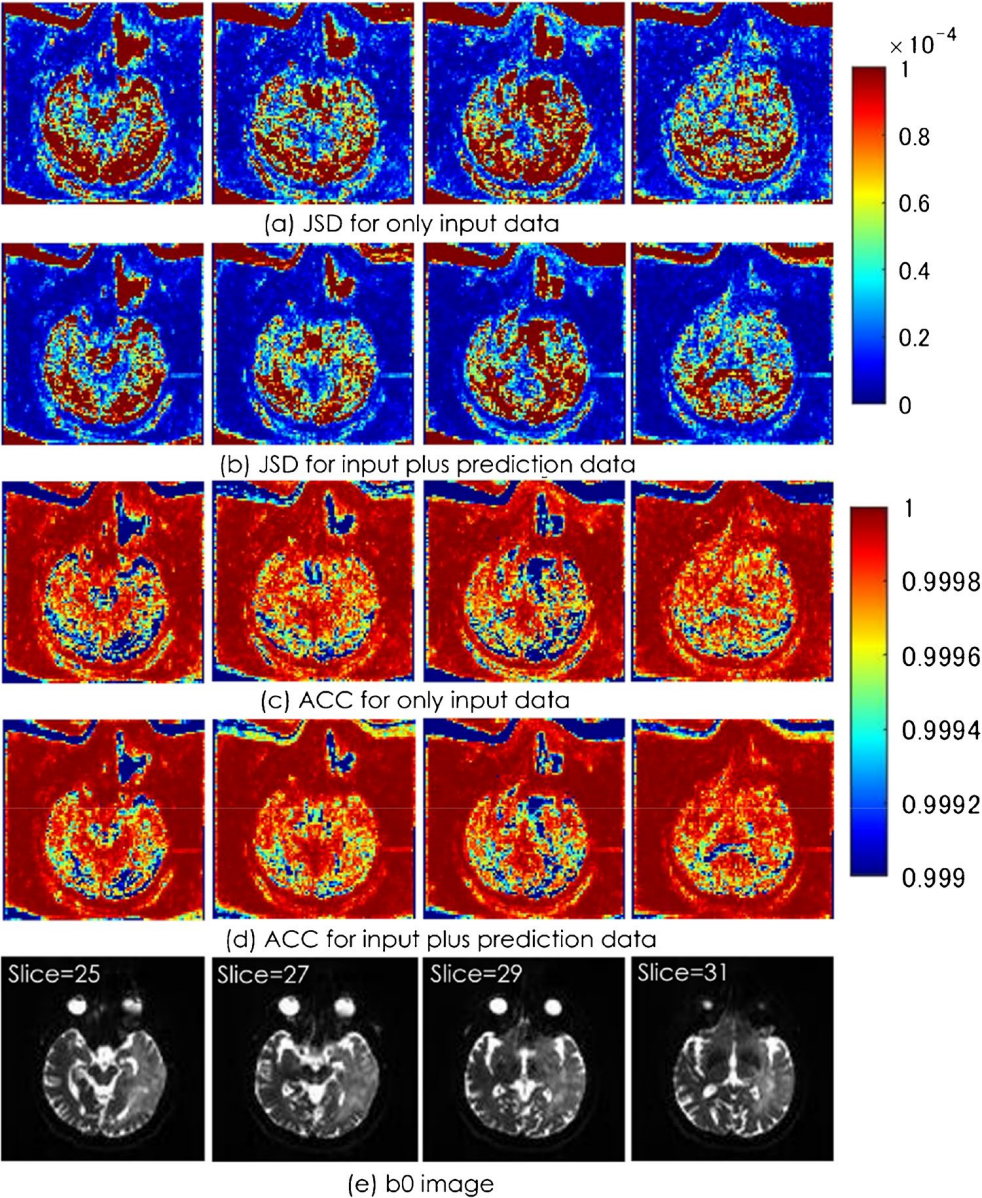
The image results from the JSD and ACC of an example case

For both the JSD and the ACC, input plus prediction data was significantly closer to the reference data than input data alone (*p* < 0.001); that is, the ODF were similar. For the JSD, the input data was 1.25*10^–4^ and the input plus prediction data was 6.75*10^–5^. For the ACC, the input data was 9.9954*10^–1^ and the input plus prediction data was 9.9974*10^–1^. Figures 8 and 9 show the results in an example case.

Input + predicted data had JSD closer to 0 and ACC closer to 1 than input data only. This showed a high degree of similarity to the diffusion profile of the reference data from the JSD and ACC definition equations. This result was the same for all individual data and overall average.

The case shown is a 75-year-old man with a primary central nervous system lymphoma in the right frontal parietal area. The closer the JSD is to 0, the higher the similarity to the reference data diffusion profile.

Overall, there was greater similarity (profile shifted to the left) with input plus prediction data (orange sticks) than with input data alone (blue sticks).

ACC is a quantitative value indicating that the closer it gets to 1, the higher the similarity. Overall similarity was better (profile shifted to the left) with input plus prediction data (orange sticks) than with input data alone (blue sticks).

ACC, angular correlation coefficient; JSD, Jensen-Shannon divergence.

The case shown is a 71-year-old man with anaplastic astrocytoma in the left temporal lobe. It can be seen that the addition of prediction data improved the quantitative values of the JSD and ACC. However, especially in ACC, even input data alone showed high similarity to reference data, so adding prediction data did not have a large effect.

ACC, angular correlation coefficient; JSD, Jensen-Shannon divergence.

### Tensor analysis of the test data

**Fig. 10:**
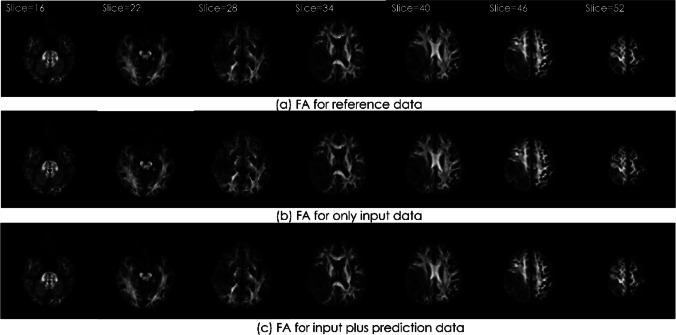
The image results from the FA of an example case

**Fig. 11:**
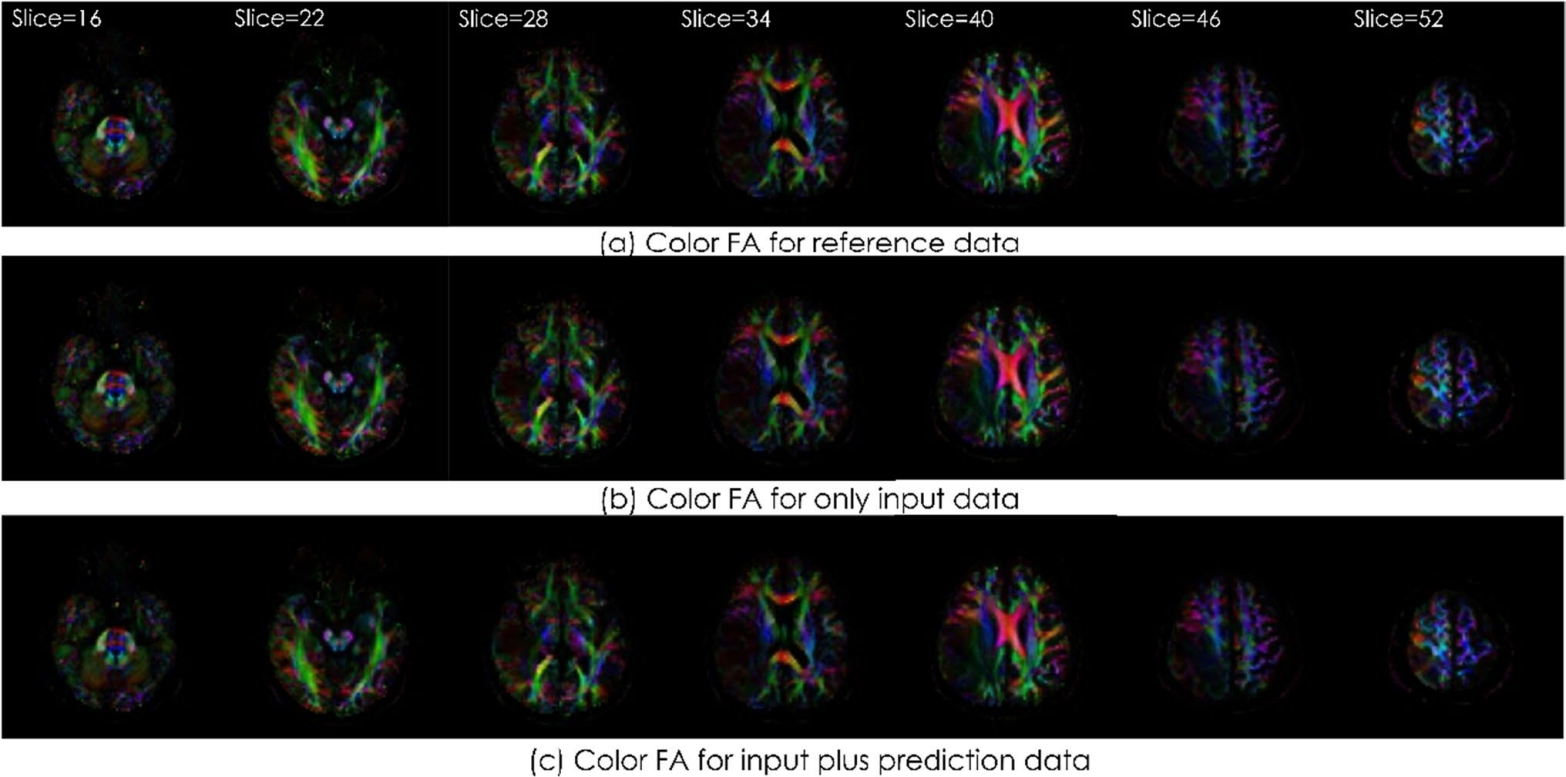
The image results from the Color FA of an example case

Example images of FA and color FA map are shown in Fig. 10 and Fig. 11. In each figure, the upper row is the images of reference data, the middle row is the images of only input data, and the lower row is the images of input plus prediction data. There was no visually obvious difference.

The case shown is a 37-year-old woman with glioblastoma in the right temporal lobe. The upper row is the images of reference data, the middle row is the images of only input data, and the lower row is the images of input plus prediction data. There was no visually obvious difference.

The case shown is a 37-year-old woman with glioblastoma in the right temporal lobe. The upper row is the images of reference data, the middle row is the images of only input data, and the lower row is the images of input plus prediction data. There was no visually obvious difference.

The average FA and SD in ROI1 was 0.552 (± 0.077) for the reference data, 0.562 (± 0.078) for the only input data, and 0.554 (± 0.080) for the input plus prediction data, respectively. The average FA and SD in ROI2 was 0.521 (± 0.044) for the reference data, 0.512 (± 0.041) for the only input data, and 0.520 (± 0.046) for the input plus prediction data, respectively. The average FA and SD in ROI3 was 0.471 (± 0.044) for the reference data, 0.470 (± 0.052) for the only input data, and 0.471 (± 0.048) for the input plus prediction data, respectively. The average FA and SD in ROI4 was 0.434 (± 0.049) for the reference data, 0.437 (± 0.050) for the only input data, and 0.437 (± 0.051) for the input plus prediction data, respectively. In ROI2, there were significant differences between reference data and only input data (p < 0.001) and between only input data and input plus prediction data (p < 0.01).

The average E1 [*µ*m/ms^2^] and SD in ROI1 was 0.664 (± 0.11) for the reference data, 0.659 (± 0.11) for the only input data, and 0.655 (± 0.11) for the input plus prediction data, respectively. The average E1 [*µ*m/ms^2^] and SD in ROI2 was 0.682 (± 0.067) for the reference data, 0.680 (± 0.066) for the only input data, and 0.680 (± 0.071) for the input plus prediction data, respectively. The average E1 [*µ*m/ms^2^] and SD in ROI3 was 0.684 (± 0.051) for the reference data, 0.683 (± 0.048) for the only input data, and 0.680 (± 0.049) for the input plus prediction data, respectively. The average E1 [*µ*m/ms^2^] and SD in ROI4 was 0.695 (± 0.057) for the reference data, 0.694 (± 0.057) for the only input data, and 0.695 (± 0.059) for the input plus prediction data, respectively. There were no significant differences among comparison conditions (Suppl. data).

**Fig. 12:**
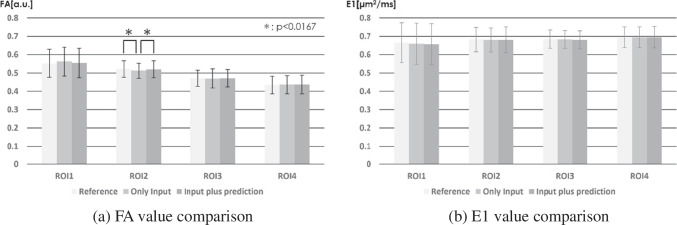
The results of ROIs measurement

The effect sizes were 0.213 and 0.164 for FA in ROI2 between Reference and Only input, and Only input and Input plus prediction, where there was a significant difference, respectively. Effect sizes in these comparisons were "small (0.1 to 0.3)”. Other effect sizes were less than 0.1 (Suppl data)(Fig. 12).

ROI1 indicates the cerebral peduncle, ROI2 indicates posterior peduncle of the internal capsule, ROI3 indicates corona radiata and ROI4 indicates semiovale center in the normal side.

In the (a) FA value comparison, significant differences occurred between only input and reference, only input and input plus prediction. In the (b) E1 value comparison, there was no significant difference.

### Tractography evaluation of the test data

For the tractography DSCs, three bundles of CST could not be visualized by QBI tractography using TractVis (right side of subject no. 3, 22, and 30). Therefore, these three were excluded from our comparison, which was made using the remaining 57 cases. The average DSC for the input data alone was 0.697 (± 0.070). The average DSC for the input plus prediction data was 0.715 (± 0.064). The DSC was significantly higher with prediction data than with input data alone (*p* < 0.05). GQI tractography using DSI Studio also failed to visualize two bundles of CST (sub no. 3, right of 30). Thus, a comparison was made using 58 samples, excluding these two cases. The average DSC for the input data alone was 0.738 (± 0.118). The average DSC for the input plus prediction data was 0.769 (± 0.091). Again, the DSC was significantly higher with prediction data than with input data alone (*p* < 0.01).

The effect size was 0.263 and 0.288 for QBI and GQI, respectively.

**Fig. 13:**
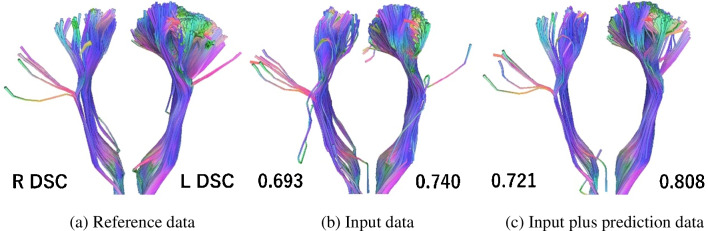
Example tractography result generated using generalized q-sampling imaging

**Fig. 14:**
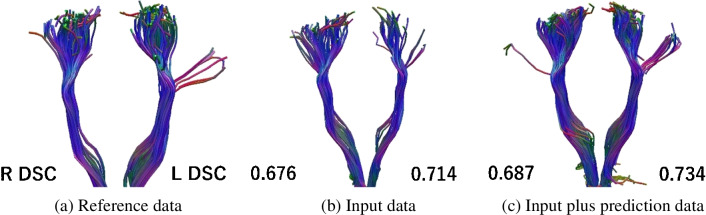
Example of a tractography result generated using Q-ball imaging

The resulting image examples shown in Fig. 13 and 14 are for the same patient. They illustrate the tendency for the DSC to decrease as a result of increases in mis-tracking and decreases in the extraction area (Figs. 13 and 14). For the case shown in Fig. 13, the DSCs of the left and right corticospinal tract with GQI were 0.693 and 0.740 for the input data, and 0.721 and 0.808 for the input plus prediction data, respectively.

The case shown is a 34-year-old man with a glioma in the left frontal lobe. The values represent the DSCs in the left and right corticospinal tracts. The DSCs of the left and right corticospinal tract were 0.693 and 0.740 for input data, and 0.721 and 0.808 for input plus prediction data, respectively.

DSC, dice similarity coefficient; L, left; R, right.

For the case shown in Fig. 14, the DSCs of the left and right CST with QBI were 0.676 and 0.714 for the input data, and 0.687 and 0.734 for the input plus prediction data, respectively.

The case shown is a 34-year-old man with a glioma in the left frontal lobe. The values represent the DSCs for the left and right corticospinal tract. The DSCs of the left and right corticospinal tract were 0.676 and 0.714 for the input data, and 0.687 and 0.734 for the input plus prediction data, respectively.

DSC, dice similarity coefficient; L, left; R, right.

An increase in the DSC values was observed in 35/57 bundles (61.4%) with QBI and 42/58 bundles (72.4%) with GQI. This indicates that the AI model produced a general improvement in the tractography visualization ability.

As for FAT, three bundles of FAT could not be visualized by QBI tractography using TractVis (both side of subject no. 21, and right side of subject no. 29). Therefore, these three were excluded from our comparison, which was made using the remaining 57 cases. The average DSC for the input data alone was 0.502 (± 0.124). The average DSC for the input plus prediction data was 0.531 (± 0.128). The DSC was significantly higher with prediction data than with input data alone (*p* < 0.001). GQI tractography using DSI Studio also failed to visualize two bundles of FAT (right side of subject no. 27). Thus, a comparison was made using 59 samples, excluding one case. The average DSC for the input data alone was 0.793 (± 0.101). The average DSC for the input plus prediction data was 0.816 (± 0.093). Again, the DSC was significantly higher with prediction data than with input data alone (*p* < 0.001).

The effect size was 0.235 and 0.240 for QBI and GQI, respectively.

An increase in the DSC values was observed in 42/57 bundles (73.7%) with QBI and 40/59 bundles (67.8%) with GQI. This indicates that the AI model produced a general improvement in the tractography visualization ability.

## Discussion

In this study, we generated unimaged DWI from imaged DWI HARDI data. Research on image generation using AI has evaluated the generation of CT from MR T1-weighted images [47], the generation of methionine positron emission tomography from contrast-enhanced T1-weighted images [48], and the generation of fluid-attenuated inversion recovery from DWI [49]. However, this is the first study to generate predicted images with the same contrast in the same series. We have shown that the second half of a HARDI dataset generated from the first half closely matches the imaged second-half data, with almost no bias in the results due to the MPG axis. Absolute similarity evaluation cannot be performed using SSIM alone, but previous research with 3D U-net on image similarity using other SSIM [50–52] suggests that the images generated by this study have a relatively high degree of similarity. However, the smoothness of the image may have caused the SSIM to show a high value, and further investigation is required. The QBI and GQI used in this study were HARDI-based datasets. HARDI-based datasets provide more information about cross-fibers than DTI, the imaging time is longer for requiring more MPG direction. While it is possible to reduce the imaging time using SMS, it is known that an increase in the SMS factor leads to a deterioration in image quality. Moreover, this type of artifact differs from classical artifacts that appear continuously within or along the slice, making interpretation challenging [31]. Our method can shorten imaging time by generating the second half of the imaged data from the first half. The generation of images in this way can reduce the incidence of artifacts caused by SMS.

DWI data is generally preprocessed for noise removal [33–35], distortion correction, and motion correction [39, 40]. Regarding body movement, the longer the imaging time, the more likely it is that the body will move from its initial position, which will lower the correction accuracy. However, this was an resting-state fMRI study, and it has been reported that 76% of the data in such studies could be collected in the first 10 min of the 20-min imaging time [53]. On this basis, it can be inferred that the longer the scan time, the lower the reliability of the data from the latter half of the scan time. With this in mind, the method presented here could effectively improve the accuracy and reliability of the imaging data through its halving of imaging times.

SSIM was used to evaluate the similarity of the original image, and JSD and ACC were used to evaluate the similarity of the diffusion profile. In this study, the ODF values generated using input plus prediction data were more similar to the ODF of the reference data than the ODF values calculated using input data alone. This was shown by the JSD and ACC values. It has been reported that 60–90% of the brain is chiasm fibers [54]. Furthermore, it has been established that MPGs of about 60 axes are required to visualize tractography using CSD [55] and that visualization performance is improved when using 64-axis data rather than 30-axis data in DTI analyses. It has further been reported that 30 is an insufficient number of MPG axes [11]. Of course, it cannot be said that 64 axes of MPG are sufficient but the effectiveness of adding images generated by this 64-axis MPG AI has been demonstrated.

We performed tensor analysis to calculate quantitative values and we compared FA and E1 using ROI measurements. No difference was visually observed in FA or color FA map (Fig. 10 and 11). Regarding ROI measurements, there was no significant difference between input plus prediction data and reference data. Although a significant difference occurred only in FA in the input data of ROI2, this result can be said to suggest the usefulness of this method.

We evaluated tractography visualization performance using QBI and GQI analysis. In both analysis algorithms, similar to the ODF study, adding the prediction data improved the value obtained using only the input data for reference. From the perspective of tractography depiction, we found that adding AI data improves the similarity of the predicted data to the reference data. A previous study that evaluated the reproducibility of QBI tractography targeting optic radiation found tractography DSCs using scan-rescan data of 0.6–0.75 [56]. The current input plus prediction data result (DSC = 0.715) for CST was within this range, suggesting that the images may be equivalent to scan-rescans.

On the other hand, the FAT result (DSC = 0.531) was out of range (0.6–0.75). This may be due to the effect of the arcuate fasciculus intersecting the FAT. As a result, we considered that FAT's DSC is overall lower than CST's DSC.

Regarding the difference in analysis algorithms, the DSCs produced by GQI analysis were superior to those from QBI. Although the DSCs were not compared, a previous study found no significant difference in the percentage of true fibers in the pyramidal tract but that the QBI calculated a superior proportion to the GQI [57]. And this report found a high proportion of false fiber, which caused the GQI's DSC to be higher than the QBI's DSC. The present authors assume that these results support the current study.

**Fig. 15:**
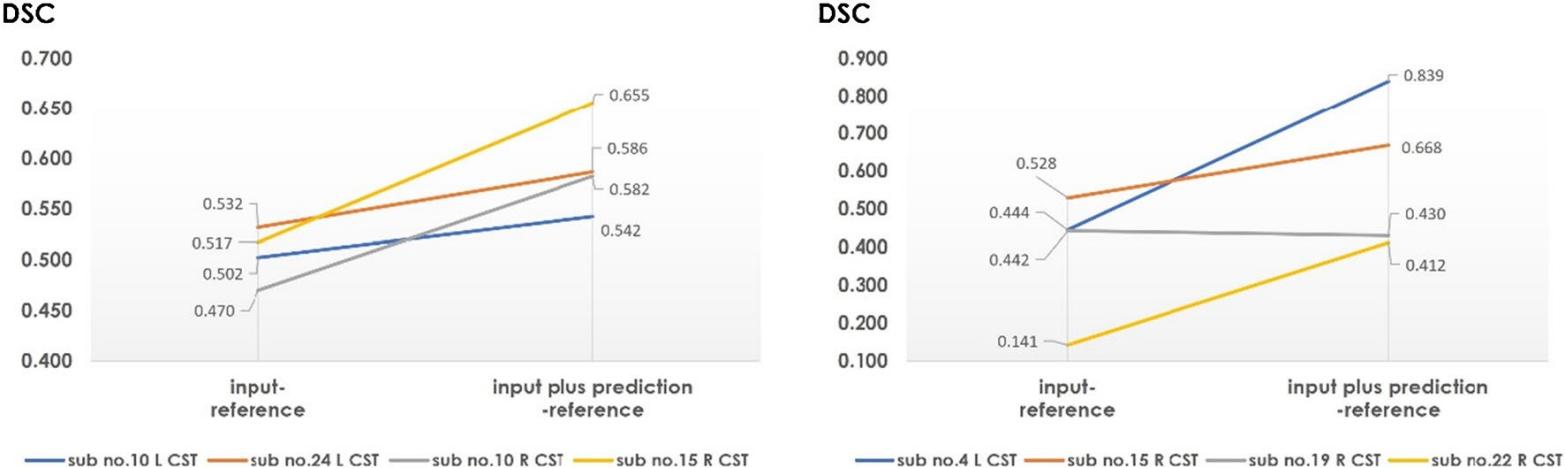
DSC improvement through the addition of prediction data in cases with DSC < 0.6 for input data alone. (**a**) The result in QBI. (**b**) The result in GQI

Regarding the improvement of CST tractography visualization ability by AI, a DSC increase of 61.4% for the QBI and 72.4% for the GQI was observed. When evaluated using the DSC classification of a previous study [54], we found that adding prediction data led to improved outcomes when the input data alone had DSC ≥ 0.6 (good or excellent), but no major improvement was seen (DSC of QBI increased by 0.013, GQI increased by 0.017). On the other hand, in one case, there was a decrease in the DSC in GQI when the DSC < 0.6, but the DSC for QBI showed an increase of 0.087 and the DSC for GQI showed an increase of 0.264 (Fig. 15).

The DSC values decreased with the addition of prediction data in a GQI case but increased in all other cases. The improvement in DSCs resulting from the addition of the prediction data was more effective when the input data DSC was lower.

DSC, dice similarity coefficient; CST, corticospinal tract; GQI, generalized q-sampling imaging; L, left; QBI, Q-ball imaging; R, right.

For the improvement of FAT tractography visualization ability by AI, a DSC increase of 73.7% for the QBI and 67.8% for the GQI was observed. Similar to the trend for CST, we found that adding predicted data led to improved results when the DSC of input data alone was 0.6 or higher (good or excellent) [53], but no significant improvement was observed (DSC of QBI increased by 0.010 and GQI increased by 0.024). On the other hand, when DSC < 0.6, the GQI DSC remained almost unchanged (0.307 to 0.300) in only one case. Regarding QBI, DSC worsened in 8 patients (average decrease of 0.07, maximum decrease of 0.172), and DSC improved in 30 patients (average increase of 0.07, maximum increase of 0.206).

This result suggests that cases with lower DSCs and lower accuracy due to disease may benefit from this approach, especially in CST. However, there were cases in which the DSC was < 0.6 even on the healthy side, so further verification is necessary.

Our study had some limitations. The generated images were only verified using one MRI machine and one type of MPG array. Also, the second 50% of the data was generated from the first 50%. Other generation ratios (e.g., generating the last 30% from the first 70%) were not attempted for comparison. Therefore, the relationship between the generation rate and image similarity was not ascertained. For the diffusion profile, only the ODF was evaluated. We had planned to test another algorithm using the FOD generated by CSD analysis but, because the test data was from brain tumor patients, it was necessary to focus only on the white matter as the ROI. The ROI was set using ss3t-CSD, which can extract white matter like even with a single unit of b-value data [58].The patient’s data for this study have many different diagnoses, it is not always the same tracts can be compared. The data set for this study was performed as part of a preoperative examination for neurosurgery, and imaging time was limited. Therefore, compared to previous studies, the resolution in this study is poorer, so the influence of partial volume effects may be stronger. In addition, although the gold standard of this tractography was used as the reference data, it is unclear whether the fiber tracing results are accurate.

## Conclusion

We developed an artificial intelligence model based on the 3D U-net architecture capable of predicting the latter 32 axes of HARDI data (MPG64 axes) from the initial 32 axes. The integration of predicted data into the input significantly improved diffusion profiles and tractography. This suggests the potential use of this approach to reduce MRI scan times by incorporating AI-generated predicted data alongside the scanned data.

## Supplementary Information

Below is the link to the electronic supplementary material.

Supplementary file1 (XLSX 51 KB)

## Data Availability

The authors confirm that the data supporting the findings of this study are available within the article and its supplementary materials.

## Abbreviations

ACC: Angular correlation coefficient
ADC: Apparent diffusion coefficient
AI: Artificial intelligence
CST: Corticospinal tract
DSC: Dice similarity coefficient
DTI: Diffusion tensor imaging
DTT: Diffusion tensor tractography
EPI: Echo planar imaging
FAT: Frontal aslant tract
FOD: Fiber orientation distribution
GQI: Generalized q-sampling imaging
HARDI: High angular resolution diffusion imaging
JSD: Jensen-Shannon Divergence
MPG: Motion-probing gradient
ODF: Orientation distribution function
QBI: Q-ball imaging
ROI: Region of interest
SMS: Simultaneous multi-slice
SSIM: Structural similarity
